# Surface morphology and mechanical properties of conventional and self-adhesive resin cements after aqueous aging

**DOI:** 10.1590/1678-7757-2017-0449

**Published:** 2018-11-08

**Authors:** Yahui Pan, Xiaodong Xu, Fangfang Sun, Xiangfeng Meng

**Affiliations:** 1 Department of Prosthodontics, Nanjing Stomatological Hospital, Medical School of Nanjing University, Nanjing, China; 2 Department of Stomatology, Northern Jiangsu People's Hospital, Yangzhou, China

**Keywords:** Resin cement, Aging, Surface property, Sorption, Solubility, Hardness

## Abstract

The stable long-term performance of resin cement under oral environmental conditions is a crucial factor to obtain a satisfactory success of the allceramic dental restoration. Objective: This study aimed at evaluating and comparing the surface morphology and mechanical property of conventional and self-adhesive resin cement after aqueous aging. Materials and Methods: Disc-shaped specimens of 3 conventional (C1: Multilink N, C2: Duolink, C3: Nexus 3) and 3 self-adhesive (S1: Multilink Speed, S2: Biscem, S3: Maxcem) types of resin cements were subjected to irradiation. After 24 h, the Knoop microhardness of each resin cement was evaluated. The specimens were immersed separately in distilled water and maintained at 37°C. A total of 5 specimens of each resin cement were collected at the following time intervals of immersion: 1, 6, 12 and 18 months. The samples were used to evaluate the Knoop parameters of microhardness, sorption and solubility. The surface morphology of the specimens after 18 months of immersion was observed by scanning electron microscopy. The sorption and solubility data were analyzed by two-way ANOVA. The Knoop microhardness was tested by the ANOVA repeated measures (P<0.05). Results: The sorption and solubility parameters of C1 and S1 exhibited significant fluctuations during the aqueous aging. The hardness of the S1 and S2 specimens decreased significantly after an 18-month water immersion. The S1, S2 and S3 specimens indicated higher filler exposure and stripping and apparent pores and cracks compared to specimens C1, C2 and C3, respectively. Conclusion: The surface of selfadhesive resin cements is more susceptible to aqueous damage than that of the conventional resin cements.

## Introduction

Bulk fractures were a crucial reason for ceramic inlay failure. ^1^ ^,^ ^2^ However, the marginal degradation was considered to be the underlying cause for these failures. ^3^ ^,^ ^4^ The bonding agent of the resin cement can lead to a loss of support for the ceramics, which produce microfractures that eventually develop into bulk fractures. ^5^ Under physiological conditions, intraoral mechanisms of sorption, hydrolysis, and dynamic fatigue may lead to polymer degradation. Walker, et al. ^6^ (2003) suggested that aqueous aging with cycling loading could increase the resin matrix fracture and the proportion of filler/resin interface fracture, which contributed to the cohesive failure of resin cement *in vivo* ^6^ . Thus, the stable long-term performance of resin cement under oral environmental conditions is a crucial factor to obtain a satisfactory success of the all-ceramic dental restoration.

At present, various self-adhesive resin cements are widely used for luting crowns, inlays, and onlays, which are made of composite, alloy, ceramic and zirconia, and fiber and titanium posts. This is due to their ability to preserve the tooth in the absence of restoration conditioning and surface treatment, ^7^ reducing the time required for the clinical procedure and technique sensitivity. In contrast to conventional resin cement, the self-adhesive resin cement contains functional monomers, namely (meth)acrylate monomers with either carboxylic acid groups, such as 4-methacryloxyethyl trimellitic anhydride (4-META), or phosphoric acid groups, like 10-methacryloxydecyl dihydrogen phosphate (MDP) ^8^ . These acid monomers can demineralize and infiltrate the tooth substrate, resulting in micromechanical retention, ^9^ ^,^ ^10^ while they can react with the tooth tissue hydroxyapatite to form the necessary chemical bond. ^11^ The concentration of acidic monomers in the self-adhesive resin cement should be considerably low to avoid excessive hydrophilicity in the final polymer, and sufficiently high to achieve an acceptable bonding to the dentin and enamel. ^12^ Following their initial mixture, the selfadhesive resin cements are fairly hydrophilic, which facilitates their wetting conditions and their adaptation to the tooth surface. Nevertheless, the materials become more hydrophobic as the acid functionality is consumed via reaction with tooth calcium ions and due to effects of various metal oxides from the ion- leachable fillers. ^8^ However, certain *in vitro* studies indicated that self-adhesive resin cements exhibit specific deficiencies. Moraes, et al. ^13^ (2011) detected the polymerization behaviors of four self-adhesive resin cements during the initial 30-min post-cure period, finding that self-adhesive resin cement had a slower polymerization rate and a lower degree of conversion in comparison with conventional resin cement under either dual- or self-cure mode. ^13^ Han, et al. ^14^ (2007) detected the degradation of self-adhesive cement surfaces following 90 days of immersion in water.

The inability of self-adhesive resin cements to control their excessive hydrophilic character can cause swelling, which may compromise both the mechanical strength as the dimensional stability. ^8^ To date, a limited number of clinical studies have reported the reliability of self-adhesive resin cements. Azevedo, et al. ^15^ (2012) showed that all indirect restorations including self-adhesive resin cement (RelyX Unicem, 3M) could be acceptable after 12 months of clinical use. *In vitro* studies conducted by Aschenbrenner, et al. ^2^ (2012) suggested that the marginal adaptation of all-ceramic MOD-inlays, luted with both dentin- and enamel- restricted cavities, by self-adhesive resin cements was successful. ^16^ In addition, the bond strength required for coronal dentin of self-adhesive resin cements has proved to be an optimal one- or two-step adhesive, ^9^ whereas the bond durability regarding glass ceramic was equivalent to the conventional resin cement. ^17^ However, these *in vivo* and *in vitro* studies have not confirmed the long-term reliability of self-adhesive resin cements under oral environmental conditions. The frequent use of additional self-adhesive resin cements has developed the requirement for extensive research regarding their long-term stability and performance under aqueous environmental conditions.

The aim of this study was to evaluate the surface morphology, and Knoop microhardness, sorption, and solubility of conventional and self-adhesive resin cements after long-term aqueous aging, and to compare their surface aging behaviors. The null hypothesis tested was that the surface morphology and hardness of self-adhesive resin cements exhibit no significant difference from the conventional resin cements after aqueous aging.

## Material and methods

### Materials

**Figure 1:**
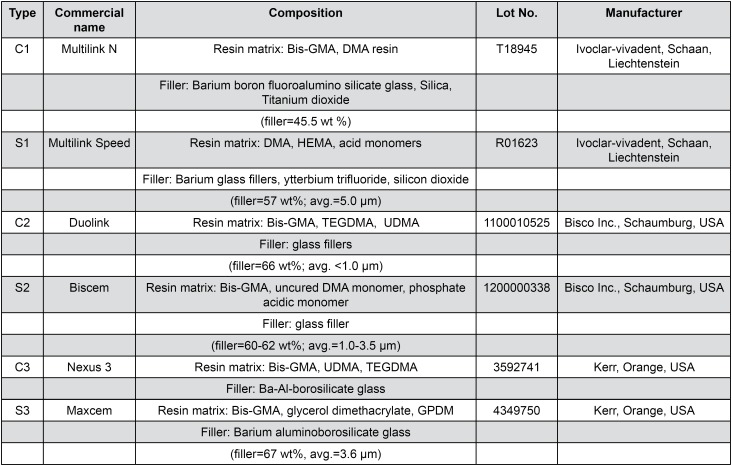
Components of the resin cements tested in this study Bis-GMA: Bisphenol A-diglycidyl methacrylate; TEGDMA: triethylene glycol dimethacrylate; DMA: dimethacrylate; HEMA: 2-hydroxyethyl methacrylate; UDMA: urethane dimethacrylate; GPDM: Glycerophosphoric acid dimethacrylate

A total of 3 pairs of conventional (C) and selfadhesive (S) resin cements (C1: Multilink N, C2: Duolink, C3: Nexus 3; S1: Multilink Speed, S2: Biscem, S3: Maxcem) were used in this study. Their composite specifications are listed in Figure 1 . Specimen preparation and Knoop microhardness measurement were conducted prior to immersion.

All the resin cements were mixed according to the manufacturers' instructions and filled into organic glass molds, which were 7 mm in diameter and 1 mm in height. A total of 2 transparent polyethylene films were placed on both sides. A glass slide was overlaid on both sides of the specimens, and slightly finger-pressed to extrude the resin cement excess. Subsequently, a single side of the mold was irradiated for 20 s with a LED dental light (800 mW/cm^2^, bluephase C8, Ivoclar Vivadent, Schaan, Liechtenstein). After irradiation, the specimens were collected from the molds. The excess cement around the specimens was removed with a scalpel blade. A total of 25 specimens were prepared for each resin cement. These specimens were kept in a light-proof container at 37°C for 24 h.

A total of 5 specimens were collected randomly from each resin cement and were evaluated by the Knoop microhardness test (HV-1000, Shanghai Metallurgical Equipment Company Ltd., Shanghai, China). The loading weight was 25 g (0.245 N) and the loading time was 15 sec. Every specimen was tested five times, and the average value (MPa) was calculated. These Knoop microhardness values prior to water immersion were used as control values.

### Knoop microhardness, sorption, and solubility measurements after immersion

At 24 h following irradiation, all specimens were placed in a silica gel desiccator (Shanghai Yetuo Instrument Company Ltd., Shanghai, China) and were stored at 37°C for 24 h. Subsequently, they were stored in the silica gel desiccator at 23°C for 1 h. The mass of these specimens was assessed on a digital balance (FA2004, Shanghai Sunny Hengping Scientific Instrument Company Ltd., Shanghai, China). This procedure was replicated to attain a constant mass (m1, μg).

A total of 20 specimens corresponding to each resin cement were divided randomly into 4 subgroups (n=5) and separately immersed in a 10 ml light-proof glass vial of distilled water, which was maintained at 37°C for the following immersion time intervals: 1, 6, 12, and 18 months. The water was changed every month.

After immersion, five specimens were collected and washed with distilled water. The specimens were dry-blotted with an absorbent paper to remove the excess of surface liquid and weighted until the balance reached a constant weight, designated as m2 (μg).

At this time point, the Knoop microhardness of these specimens was tested according to the test conditions previously mentioned.

Finally, these specimens were reconditioned according to the constant mass, following the aforementioned desiccation procedure one more time. The constant mass was marked as m3 (μg).

In accordance with the ISO 4049 specification ^18^ , values for the sorption (Wsp) and the solubility (Wsl) at specific times were calculated using the following equations, respectively:

(1) Wsp=[m2–m3]÷V

(2) Wsl=[m1–m3]÷V

Where m1 is the initial mass before immersion; m2 is the saturated mass at a specific time; m3 is the final mass at a specific time; V is the volume of the specimen.

### Surface morphology of the specimen after 18 months of water immersion

After 18 months of immersion, the surface morphology of specimens after the measurement of sorption and solubility was observed using scanning electron microscopy (SEM, S-4800, Hitachi Ltd, Tokyo, Japan).

### Statistical analysis

The mean values and standard deviations were calculated for each test group. The data were analyzed by SPSS (Version 20.0, SPSS Inc., Chicago, Illinois, USA). Sorption and solubility were analyzed by twoway ANOVA (resin cements, immersion time), and one-way ANOVA and SNK tests were used as a further comparison. The repeated measurement was used for Knoop hardness. The significance was set at 0.05 (P<0.05).

## Results

### Knoop microhardness

Changes in the microhardness levels of all resin cements during the total period of water immersion are shown in Table 1 . Microhardness values prior to immersion were used as a baseline. The microhardness of 3 conventional resin cements exhibited no significant change during the total period of water immersion. However, the three selfadhesive resin cements exhibited different changing patterns regarding microhardness during the total water immersion period. The microhardness value of S1 decreased significantly only after 18 months of immersion, whereas that of S2 decreased gradually and that of S3 exhibited no significant decrease during the entire immersion process.

**Table 1:** Mean (standard deviation) Knoop microhardness of conventional and self-adhesive resin cements

Material	Immersion time				
	24 h	1 mon	6 mon	12 mon	18 mon
C1	35.25(0.74)^a^	34.92(0.16)^a^	34.68(3.95)^a^	33.59(1.82)^a^	33.31(0.83)^a^
C2	36.19(1.48)^a^	36.60(3.88)^a^	36.84(1.05)^a^	36.50(4.18)^a^	35.80(2.56)^a^
C3	29.05(0.98)^a^	29.26(3.50)^a^	29.84(3.06)^a^	29.82(3.69)^a^	29.18(3.29)^a^
S1	38.63(4.27)^a^	41.14(2.59)^a^	42.21(1.78)^a^	37.56(2.53)^a^	21.95(1.03)^b^
S2	22.76(1.16)^a^	21.28(1.19)^b^	18.89(1.28)^c^	16.89(1.03)^d^	16.91(0.82)^d^
S3	18.63(2.89)^a^	21.81(1.56)^b^	25.60(2.39)^c^	24.57(2.20)^bc^	18.00(2.42)^a^

In the conventional resin cement, no significant differences were noted when its values were compared with the corresponding ones prior to the immersion. In self-adhesive resin cement, the same superscript indicates no significant differences compared with the values prior to immersion (24 h) (P<0.05)

### Sorption and solubility

**Figure 2:**
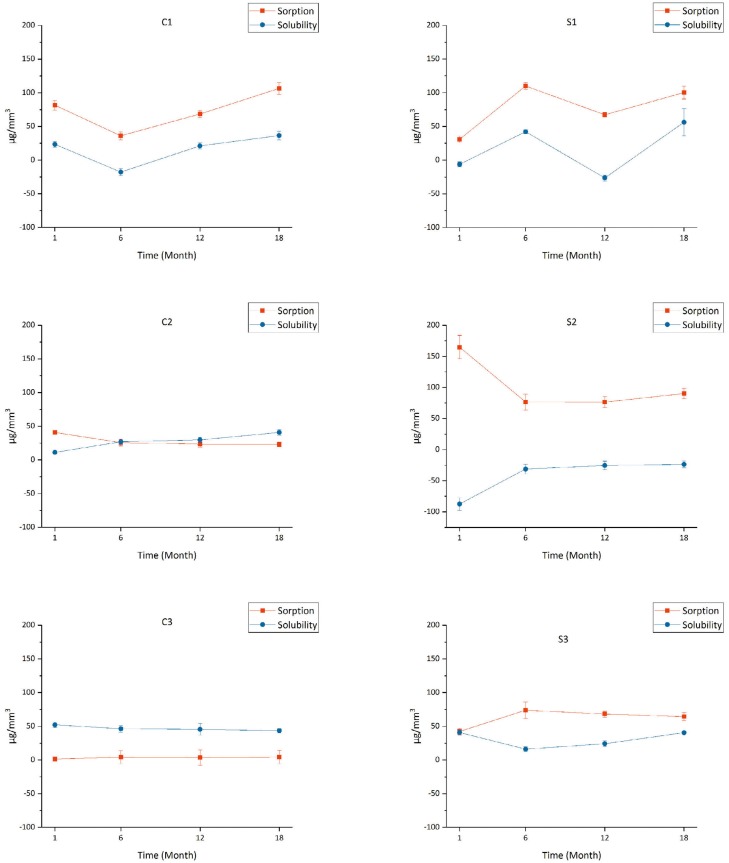
Sorption and solubility of conventional and self-adhesive resin cements during the total period of water immersion

Two-way ANOVA showed that the sorption and solubility were significantly influenced by time (p<0.001) and materials (p<0.001), and by the interaction between them (p<0.001). Changes in the sorption and solubility values of all resin cements during the total period of aqueous aging are graphically presented in Figure 2 . During 18 months of aqueous aging, the sorption and solubility of C1 and S1 indicated fluctuating changes, while the sorption and solubility of C2, C3, and S3 exhibited no apparent fluctuations. In the first 6-month period of aqueous aging, the sorption and solubility of S2 showed a significant fluctuating change. Following this time period, the change trend was stable.

### Surface morphology observation

**Figure 3:**
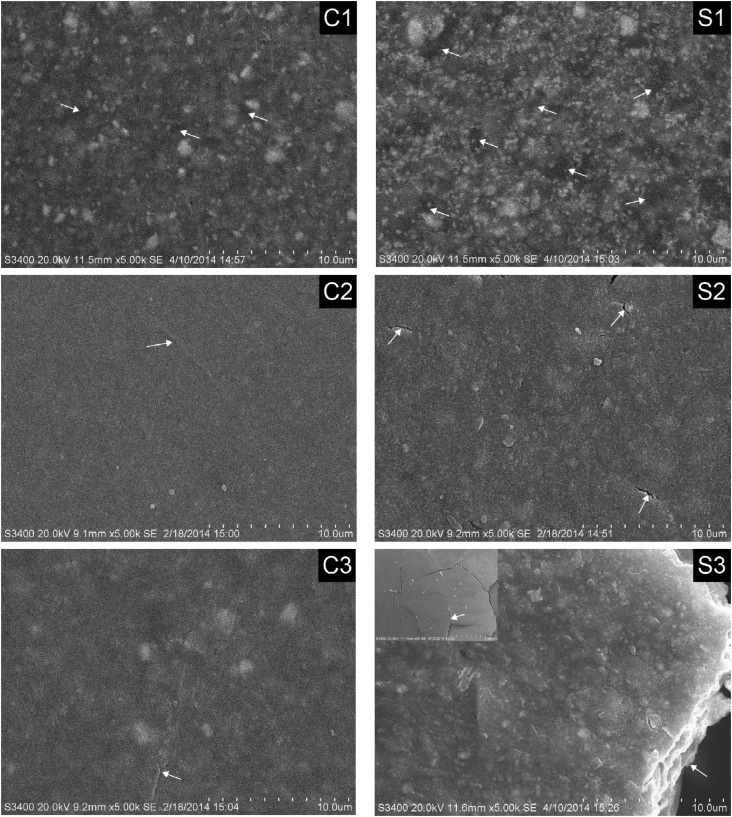
Scanning electron microscopy (SEM) photographs of all resin cements, after 18 months of water immersion. The exposed fillers, cracks, and voids can be observed in each photograph and are marked with white arrows

The surface morphology of the six resin cements after 18 months of water immersion is shown in Figure 3 . S1 exhibited higher levels of filler exposure and stripping compared with C1, while S2 and S3 had apparent cracks and/or pores compared to C2 and C3. The specimen S3 was completely fractured.

## Discussion

The manufacturers of several materials often do not entirely disclose details of material composition and, as a result, self-adhesive resin cements notably lean on acidic monomers that impose formulation stability complications. In clinical practice, the aging of resin cement may lead to restorative failure. With the exception of occlusion and abrasion, the sorption and solubility are significant parameters that should be considered for the preservation of resin cements.

Polydimethacrylate resins such as resin cement are glassy polymers. The water sorption in glassy polymers is generally described by a dual-mode theory, which assumes that the amount of sorbed molecules consists of two populations. ^19^ ^,^ ^20^ The first molecular population follows the ordinary Henry's law and the second one is trapped in polymer microvoids, following the Langmuir isotherm. This phenomenon is clearly described by the free volume theory, which suggests that glassy polymers generally have a nonequilibrium liquid structure and contain an equilibrium hole-free volume defined by Henry's law, as well as an extra non-equilibrium hole-free volume, frozen into the micro-voids, that is described by the Langmuir's isotherm. ^21^ ^,^ ^22^

In this study, C2 and C3 exhibited no significant change of sorption and solubility during the aqueous aging process and their solubility values were positive, suggesting that C2 and C3 could absorb water and elute non-reacted monomers in an aqueous environment according to Henry's law. In addition, water molecules would occupy the available space, such as microvoids and morphological defects and, consequently, their polymer construction would exert no significant change. This was confirmed by their surface hardness and surface morphology after 18 months of aqueous aging.

An increase in the free space should lead to an increased sorption, while the solubility values indicated the changes noted in the free space to some extent. While C1 showed a wavy change of sorption and solubility during aqueous aging, its solubility value was negative, even at 6 months of aqueous aging. This indicates that, in addition to Henry's law, Langmuir's sorption played a significant role during aqueous aging. The sorption occurred by the successive binding interactions with the hydrophilic groups that formed hydrogen bonds. ^23^ This suggested that C1 could be more hydrophilic when compared to C2 and C3. Although the surface hardness of C1 exhibited no significant decrease, the surface morphology indicated the evidence of filler exposure and stripping.

S1 exhibited a significantly wavy change of sorption and solubility compared to C1. In addition to the hydrophilic acid-monomer, according to the information provided by the manufacturer the S1 specimen contained HEMA, which is a mono-vinyl monomer commonly used as the polymerizable component and as a hydrophilic primer in adhesive resins. ^24^ HEMA may further enlarge the polymer network, resulting in the additional formation of microvoids with increased uptake of "free" water. ^25^ ^,^ ^26^ The more hydrophilic S1 indicated additional filler exposure and stripping compared to C1, which resulted in the decrease of surface hardness after 18 months of aqueous aging, since the hardness was significantly affected by the filler volume. S1 revealed negative values, meaning a loss of weight, which showed the same results as the previous study. ^27^

S2 indicated a significant fluctuating change of sorption and solubility in the first 6 months of aqueous aging in comparison with C2. However, the solubility value of S2 was negative during the total period of aqueous aging. It was suggested that the transfer of water molecules occurred from an absorbed state to a bound state, which was dispersed into the polymer matrix and acted as a plasticizer that caused the polymer swelling. This could explain the S2 surface hardness decrease after 18 months of aqueous aging. In addition, the plasticization of water might damage the structure of the resin matrix and produce additional surface pores and cracks during aqueous aging.

The change in the parameters of sorption and solubility of S3 were similar to those of C3, although negative solubility was not observed. However, the S3 containing acid-monomer exhibited higher sorption value compared to the C3. The water sorption did not affect the surface hardness, although it damaged the structure of the resin matrix, which resulted in the complete fracture of specimen S3. Previous studies have shown that S3 exhibited poor bond durability with dentine, and the bond failure of S3 and dentine was 100% in adhesive fractures.^10,23,28^

In this study, the surface morphology of the conventional resin cements indicated higher integrity, while the self-adhesive resin cements exhibited additional filler exposure and striping, as well as pores, grooves, cracks and even complete specimen fracture, as determined by SEM. Thus, the hypothesis that the water aging behavior of self-adhesive resin cements exerts no significant effects from that of conventional resin cements must be rejected.

Marginal integrity and bonding effectiveness have been reported to be the most important factors affecting the restoration longevity. ^29^ ^,^ ^30^ The cracking and filler stripping of resin cements may lead to marginal fracture and microleakage, which may further influence the survival rate of indirect restorations. Therefore, clinical trials with longer observation periods are required to confirm the data collected from this investigation.

## Conclusions

Within the limitations of this *in vitro* study, we concluded the self-adhesive resin cement is more susceptible to water aging in comparison with the conventional resin cement.
